# Genotyping of *Coxiella burnetii* in sheep and goat abortion samples

**DOI:** 10.1186/s12866-018-1353-y

**Published:** 2018-12-04

**Authors:** Dimosthenis Chochlakis, Ana Sofia Santos, Nektarios D. Giadinis, Dimitrios Papadopoulos, Leonidas Boubaris, Emmanouil Kalaitzakis, Anna Psaroulaki, Spyridon K. Kritas, Evanthia I. Petridou

**Affiliations:** 1 Department of Clinical Microbiology and Microbial Pathogenesis, School of Medicine, University of Crete, Voutes-Staurakia, 71110 Heraklion, Crete Greece; 2 Centre for Vectors and Infectious Diseases Research, National Institute of Health Dr. Ricardo Jorge, Águas de Moura, Portugal; 3 Clinic of Farm Animals, Faculty of Veterinary Medicine, School of Health Sciences, Aristotle University of Thessaloniki, Thessaloniki, Greece; 4 Laboratory of Microbiology and Infectious Diseases, Faculty of Veterinary Medicine, School of Health Sciences, Aristotle University of Thessaloniki, Thessaloniki, Greece; 5 Unit of Zoonoses, Laboratory of Clinical Microbiology and Microbial Pathogenesis, Faculty of Medicine, University of Crete, P.O. Box: 1393, 71110 Heraklion, Crete Greece

**Keywords:** Small ruminants, Abortion, *Coxiella burnetii*, MLVA, MST

## Abstract

**Background:**

Q fever, caused by *Coxiella burnetii*, is a zoonosis that presents a worldwide distribution and affects both humans and animals. The route of dispersal of the pathogen by ruminants into the environment usually involves stages of abortion and parturition, nevertheless the agent can, also, be detected in other animal samples. Therefore it is considered as important in terms of proper diagnosis, as well as, for epidemiology and surveillance purposes, to genotype the pathogen. The aim of the current study was to investigate the presence of different genotypes of the agent in animals that had suffered from abortion during a two-year survey in Greece.

**Results:**

Sixty nine tissue samples (37 stomach contents, 11 liver samples, 21 cotyledons) were collected from 59 abortion cases in sheep (*N* = 45) and goats (*N* = 14) from 65 farms at eight different areas of Greece. Samples were screened by qPCR and positive ones were further genotyped using a 10-locus multiple loci (ms 1, 3, 7, 12, 20, 21, 22, 26, 30 and 36) variable number of tandem repeat analysis (MLVA) method.

Three genotypes were identified in sheep (A, B, C). Samples representing each of the obtained MLVA profile were further used for MST genotyping. Ten spacers (Cox 2, 5, 6, 18, 20, 22, 37, 51, 56 and 57) were amplified. A close relatedness among the identified MLVA genotypes was confirmed since they all belonged to MST group 32.

**Conclusions:**

The current study introduces into the aspect of genotyping of *C. burnetii* in Greece. Further studies are needed to explore the presence of more genotypes, to associate the genotypes circulating in the animal and tick population with those causing human disease in order to further expand on the epidemiological aspects of the pathogen.

**Electronic supplementary material:**

The online version of this article (10.1186/s12866-018-1353-y) contains supplementary material, which is available to authorized users.

## Background

Q fever, caused by the pathogen *Coxiella burnetii,* is a zoonosis with a worldwide distribution [1]. The presence of the pathogen has been reported in a great variety of animals including wild and domestic mammals (goats, sheep, cows, buffaloes and potentially other dairy ruminants, more rarely dogs and cats). The agent has, also, been detected in ticks, although there is still controversy on whether these arthropods can act both as reservoirs for maintaining *C. burnetii* in nature and as vectors for transmitting it to humans.

Domestic ruminants (particularly sheep, goats, and cattle) are often asymptomatic carriers of the pathogen or express mild clinical manifestations associated with abortion, stillbirth, placentitis, endometritis and infertility [2–4]. These animals, either ill or healthy (carriers), are considered the major source of infection to humans [1]. In fact, a vast number of bacteria can be shed by domestic ruminants through milk, faeces, vaginal secretions and mostly placenta and birth products. *Coxiella burnetii* spores are highly infectious and stable under environmental conditions and are easily dispersed by airflow, that is why the main route of infection is considered to be inhalation of contaminated aerosols or dust [5]. Spores can, also, be spread several kilometers away from the primary infection source through wind, raising the latter as a potential player for bacterial dispersal [6].

It is of great importance to be able to trace back the sources of infection and to characterize the strains of the pathogen present in certain areas, both for epidemiological and for public health reasons (for example, surveillance purposes and trace of Q fever outbreaks). The most up to date event that leads towards the necessity of tracing and surveying the source of infection was the outbreak that took place in the Netherlands (2007–2010). This event has drawn further attention on the study of the pathogen even in countries where a low prevalence of the disease was usually recorded, as was the case of the Netherlands until the outbreak episodes [1, 7]. To achieve such a demanding goal, the most reliable way is to use molecular tools for the genotypic characterization of *C. burnetii* in order to evaluate the epidemiological link between the source of the outbreak and human and/or animal cases. The ultimate goal in any case is to establish control measures with respect to hosts involved in the life cycle of the pathogen in order to minimize or even just survey its dispersal.

A number of different molecular typing methods have been used to achieve the above mentioned goals including, restriction fragment length polymorphism (RFLP) in combination with pulse field gel electrophoresis (PFGE) [8, 9], multi-locus variable number of tandem repeats analysis (MLVA; more than 20 publications following the 1st one in 2006 [10]), multispacer sequence typing (MST) [11], and single nucleotide polymorphism (SNP) [12, 13]. Of the mentioned techniques, MLVA and MST have proved to be reliable, reproducible, and present a high discriminatory power. They do not require cultivation of the pathogen, which not only is it difficult to achieve but, also, obliges Biosafety Level (BSL) 3 facilities, while there is always the possibility of isolation failure due to contamination (especially in abortion samples). As far as MLVA is concerned, it is now considered as the reference method for a number of other pathogens: *Mycobacterium tuberculosis* [14], *Bacillus anthracis* [15], and *Yersinia pestis* [16]*. MLVA* is particularly useful in cases where there is a need for a deeper study of new genome sequences [17, 18].

In Greece, despite the fact that reporting of Q fever should be mandatory, no more than 40 human cases are recorded annually at the Hellenic Center for Disease and Control, making its incidence possibly underestimated. Moreover, the absence of *C. burnetii* routine testing of animals of veterinary interest, leads to the conclusion that very few animal cases are recorded on an annual basis. A number of studies have been carried out in Greece during the last 20 years in humans in order to document the prevalence of the disease, its potential complications, describe the difference in the kinetics of the antibodies between acute and chronic Q fever, identify possible proteins of the pathogen involved in the disease and their potential virulence, etc.; nevertheless, there is no information on the genotypic diversity of the pathogen circulating in the country (at the discussion section we comment further on the studies carried out in the country so far).

In the current study, *C. burnetii* DNA obtained from small ruminant abortion samples was genotyped. We introduce the use of MLVA and of MST in the study of the genetic diversity of the pathogen that is circulating in the country, as a first step to establish the link with potential sources of human infection.

## Methods

### Sampling

**Fig. 1:**
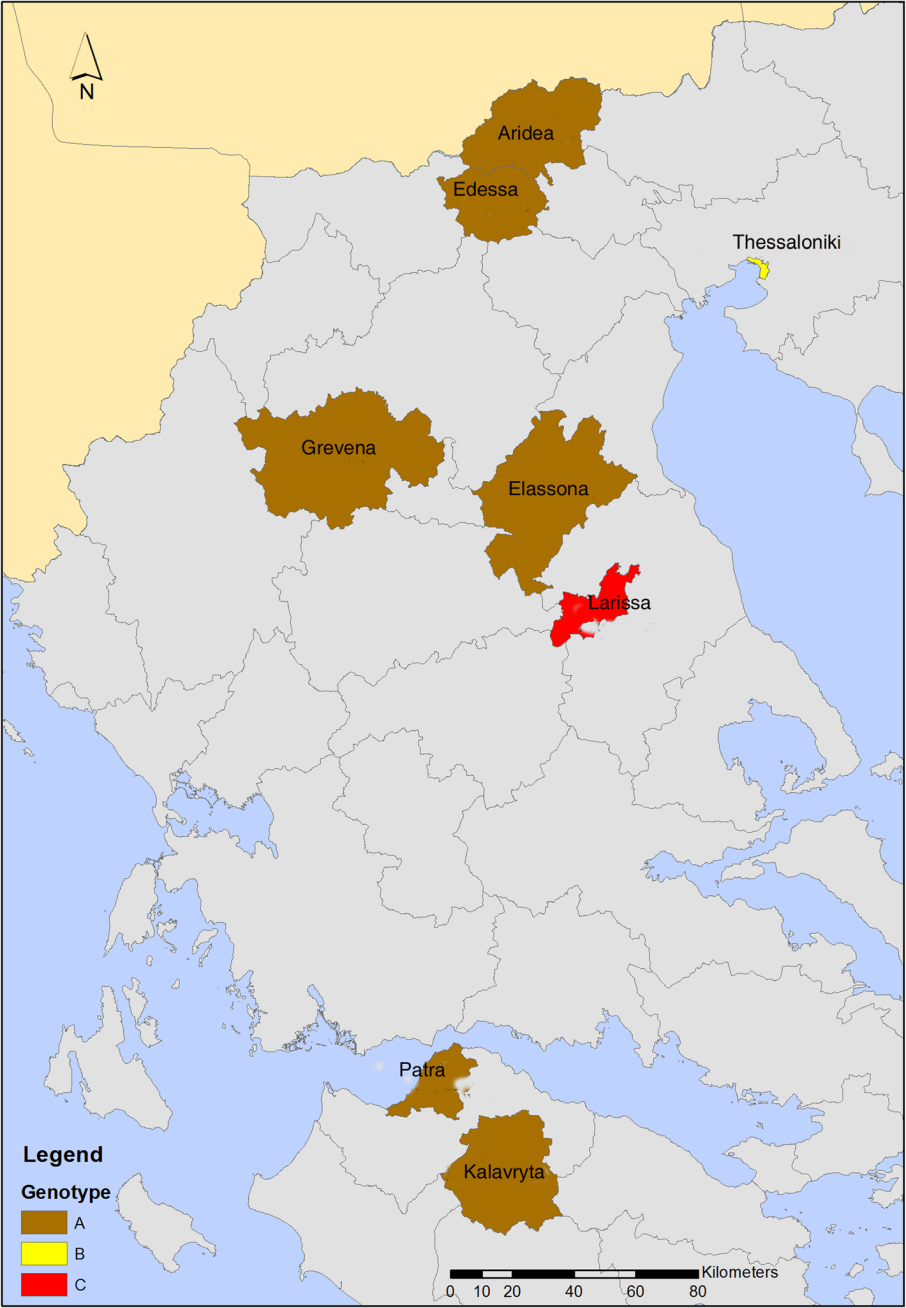
Geographical distribution of the genotypes (A, B, C) detected in the animals (sheep) tested positive by qPCR

Samples were collected from 59 abortion episodes that occurred in sheep (*N* = 45) and goats (*N* = 11) from December 2014 to November 2016. The study enrolled 65 farms situated in middle, north, northeastern and northwestern Greece (Fig. 1). A total of 69 samples consisting of stomach content (*N* = 45), liver samples (*N* = 11) and cotyledons (*N* = 14), were obtained collected immediately after the abortion, packaged with ice and transported to the Farm Animal Clinic for examination.

### Molecular analysis

DNA extraction was performed at the Clinic of Farm Animals, Faculty of Veterinary Medicine, School of Health Sciences, Aristotle University of Thessaloniki, using the QIAamp Tissue kit (QIAGEN Gmbh, Hilden, Germany). The concentration of extracted DNA was measured as the optical density at 260 nm using the Nanodrop 2000 photometer (Thermo Scientific). DNA samples were stored at -20 °C until further analysis. The initial qPCR screening of the samples and the MLVA genotyping were performed at the unit of Zoonoses of the Department of Clinical Microbiology and Microbial Pathogenesis (National Reference center for tick-borne pathogens), School of Medicine, University of Crete. The MST genotyping was carried out at the Center for Vectors and Infectious Disease Reserach, National Institute of Health Doutor Ricardo Jorge, Portugal.

### Real-time polymerase chain reaction (qPCR)

To screen for the presence of *C. burnetii* DNA a qPCR was performed targeting the repeated sequence *IS1111* [19]. The 20 μl reaction consisted of mastermix (Bio-Rad), 0.4 μM of each primer, 0.2 μM of probe and 2.5 μl of DNA sample. Amplification was carried out in a CFX96 C1000 Real-time PCR (BioRad), under the following conditions: one cycle at 95 °C for 180 s, 40 cycles at 95 °C for 10s and 55 °C for 30s. Results were generated with CFX Manager Software v. 1.6 (BioRad). Samples showing cycle threshold (Ct) values of 31 or lower for *C. burnetii IS1111* qPCR assays were considered positive, according to an already described procedure [20]. DNA extracted from a Nine Mile strain (RSA 493) strain, which is maintained in culture (Vero cells) at the laboratory, was used as a positive control and double distilled water was used as a negative one.

### Multiple-locus variable-number tandem repeat analysis (MLVA)

All *C. burnetii* positive samples were further used for MLVA genotyping. Ten different loci (ms 1, 3, 7, 12, 20, 21, 22, 26, 30 and 36) were selected for DNA amplification, as previously described [10, 21]. Nine Mile strain (RSA 493) for which the expected MLVA pattern is known, was used as a reference control strain to assist with the interpretation and the estimation of the number of repeat units. Following gel electrophoresis, the number of repeats in each marker was determined by extrapolating the sizes of the reference strain from those obtained from our samples. According to the established consensus, the genotype of the Nine Mile strain is now designated as 4–7–8-8-15-6-6-4-12-4 for markers *ms01*, *ms03*, *ms07*, *ms12*, ms20, *ms21*, *ms22*, *ms26*, *ms30* and *ms36*, respectively. Differences in PCR products were analysed using the Alpha View software v. 3 (Alpha Innotech). MLVA tools and databases of several organisms were accessed over the website http://mlva.u-psud.fr/mlvav4/genotyping/view.php. This database was made available in 2014 aggregating mainly data published in 2006 by Arricau-Bouvery et al. (“*C. burnetii* 2007 Orsay” database), data provided by Kinga Sulyok, Miklós Gyuranecz and col., Institute for Veterinary Medical Research, Budapest, Hungary (“*C. burnetii* 2014” Hungary database) and data produced since 2007 by Jeroen Tilburg et al. (“*C. burnetii* 2014 Nijmegen” database).

### Multi-spacer sequence typing (MST)

**Fig. 2:**
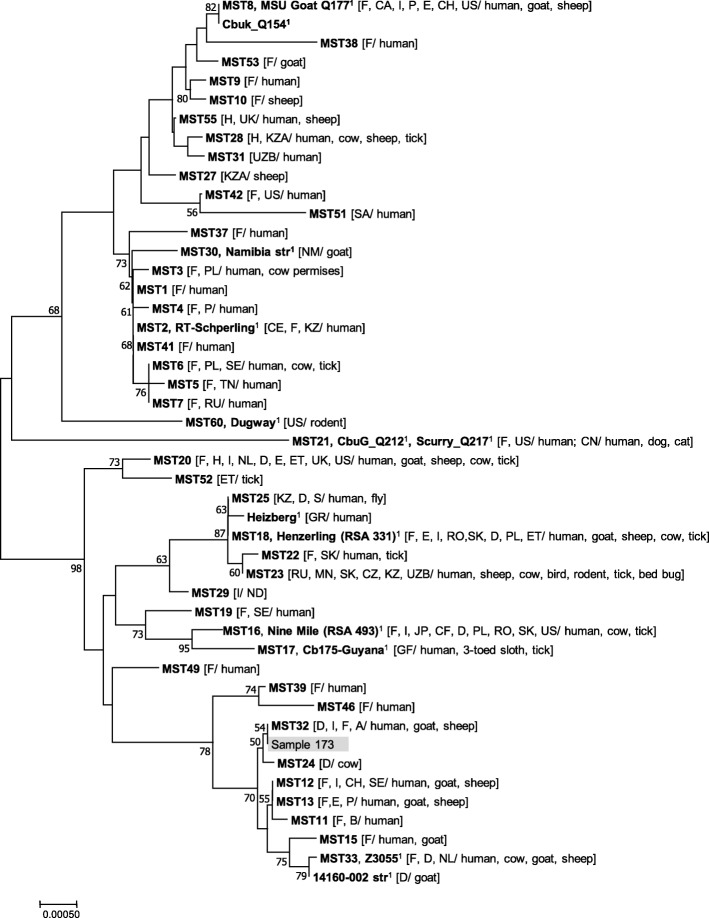
The placement of the *C. burnetii* strain detected in this study (represented by sample 173, highlighted in grey) with the known MST genotypes. The analysis was performed using MEGA v.7 software (Kumar et al., 2016 [23]) using the Neighbor-joining method (Maximum Composite Likelihood method) with 1000 replicates. Bootstrap values > 50 are shown. The scale bar represents the number of nucleotide substitutions per site. Host and geographic origin of *C. burnetii* genotypes are provided, according to open source databases and previous publications (listed in Additional file 1: Table S1). Country codes used: CE-Republic of Crimea; CF-Central African Republic; CH-Switzerland; CZ-Czech Republic; D-Germany; E-Spain; ET-Ethiopia; F-France; GF-French Guiana; GR-Greece; H-Hungary; I-Italy; JP-Japan; KZ-Kazakhstan; MN-Mongolia; NL- Netherlands; NM-Namibia; P-Portugal; PL-Poland; RO-Romania; RU-Russia; S-Sweden; SA-Saudi Arabia; SE-Senegal; SK-Slovakia; TN- Tunisia; UK-United kingdom; UKR-Ukraine; US-United States; UZB-Uzbekistan. ^1^Reference strains: MSU Goat Q177 (Genbank accession number CP18150); Cbuk_Q154 (access no CP001020); Heizberg (access no CP014561); 14,160–002 str (access no CP014836); Namibia str (access no CP007555); Dugway (access no CP000733); CbuG_Q212 (access no CP001019); Scurry_Q217 (access no CP014565); Henzerling (RSA 331) (access no CP000890); Nine Mile (RSA 493) (access no AE016828.);Cb175-Guyana (access no HG825990); Z3055 (access no LK937696); RT-Schperling (access no CP014563)

Samples representing each of the obtained MLVA profile were used for MST genotyping. In order to achieve that, ten different spacers (Cox 2, 5, 6, 18, 20, 22, 37, 51, 56 and 57) of the *C. burnetii* genome were amplified, as described elsewhere [11, 22]. Conventional PCRs were performed in 50-μl reaction using 1× FastStart Master Mix (Roche Diagnostics), containing 0.5 μM of amplification primers and 5 μl of DNA sample. After amplification purification and sequencing, the forward and reverse sequences generated for each of the 10 loci were aligned on the DNAStar sequence analysis software. The MST group was then identified using a web-based MST database (http://ifr48.timone.univ-mrs.fr/mst/coxiella_burnetii// mst/coxiella_burnetii/strains.html). In order to integrate the *C. burnetii* DNA detected, a phylogenetic analysis was performed using the MEGA v.7 software [23] using concatenated sequences of most of the known MST genotypes. The evolutionary distances were inferred using the Neighbor-Joining method (computing the Maximum Composite Likelihood method) and expressed in the number of base substitutions per site by pair wise comparison of 48 nucleotide sequences (Fig. 2). The eco-epidemiological context of MST genotypes, i.e. information on geographical distribution, occurrence and affected animal host, was compiled from the open-source MST database and from several published papers.

## Results

Of the 69 samples tested, 11 (15.9%) were positive by qPCR and were further genotyped by MLVA. All samples originated from sheep abortion cases that occurred in 11 farms located in eight different regions (Table 1 and Fig. 1). Attempts to genotype two samples with higher Cq values were unsuccessful probably due to the lower *C. burnetii* DNA content. Furthermore, we failed to amplify locus *ms26* despite repetitive attempts. Three different genotypes were detected in 11 samples: “A” was the most common and was detected in nine samples; “B” and “C” were detected in one sample each. Genotype B differed at the size of a single locus (*ms22*) only, compared to most prevalent genotype A, by a number of 33 bp, while genotype C differed at the size of three loci (*ms22, ms30* and *ms36*) by a size of 66 bp, 18 bp and 45 bp respectively, when compared to the most prevalence genotype A (Table 1). The dispersal of genotype A did not follow any particular pattern, since it was detected both in northern, central and in southern areas of the country. These genotypes represented unique MLVA profiles when compared to those deposited in open-access banks (http://mlva.u-psud.fr/MLVAnet/spip.php?rubrique50, http://microbesgenotyping.i2bc.paris-saclay.fr/databases/).

**Table 1:** Amplification of 10 loci from panel A as this has been established (http://mlva.u-psud.fr//MLVAnet/spip.php?rubrique50)

ID	Host	Source	Location	Year	Ct	MLVA											MST type
						Loci targeted										Type	
						*ms01*	*ms03*	*ms07*	*ms12*	*ms20*	*ms21*	*ms22*	*ms26*	*ms30*	*ms36*		
27	Sheep	Stomach content	Grevena	2015	27.1	0	0	0	-2	3	0	−7	N/A	0	3	A	
45/2	Sheep	Stomach content	Elassona	2015	28.6	0	0	0	−2	3	0	−7	N/A	0	3	A	
85/1	Sheep	Cotyledon	Kalavryta	2014	29.9	0	0	0	−2	3	0	−7	N/A	0	3	A	
85/1	Sheep	Stomach content	Kalavryta	2014	27.4	0	0	0	−2	3	0	−7	N/A	0	3	A	
92	Sheep	Liver	Edessa	2015	28.3	0	0	0	−2	3	0	−7	N/A	0	3	A	
113	Sheep	Stomach content	Thessalonika	2015	23.5	0	0	0	−2	3	0	−4	N/A	0	3	B	32
152/2	Sheep	Cotyledon	Patra	2016	29.1	0	0	0	−2	3	0	−7	N/A	0	3	A	
156/B	Sheep	Stomach content	Aridea	2016	28.4	0	0	0	−2	3	0	−7	N/A	0	3	A	
173	Sheep	Liver	Elassona	2016	24.6	0	0	0	−2	3	0	−7	N/A	0	3	A	32
173	Sheep	Stomach content	Elassona	2016	23.7	0	0	0	−2	3	0	−7	N/A	0	3	A	32
14/1	Sheep	Cotyledon	Larissa	2017	24.2	0	0	0	−2	3	0	1	N/A	−1	9	C	32

*ID* Sample Identification number, *Ct* Real-time PCR cycle threshold, *N/A* not determined DNA. The genotypic designation of Nine Mile (4–7–8-8-15-6-6-4-12-4, for the corresponding loci) was used to compare our findings. Where “0”, we ended up with the same band size during the MLVA typing; differences either these are “-” or “+” correspond to differences in number of repeats among our samples and Nine Mile. MLVA and MST genotypes were confirmed in *C. burnetii* 2014 cooperative database (http://mlva.u-psud.fr/mlvav4/genotyping/view.php) (http://ifr48.timone.univ-mrs.fr/mst/coxiella_burnetii/strains.html)

To complement the molecular analysis and to try to find a match with previous detected *C. burnetii* genotypes, one sample representing each of the MLVA profile was, also, used for MST typing. This is a less discriminatory method however, it is broadly used and has a standardized nomenclature allowing easy comparison of results among laboratories. The obtained results, confirmed the close relatedness among the MLVA genotypes A, B, C since they were all identified as belonging to MST group 32. The allele codes found were 3–5–1-6-5-4-5-12-3-2 for spacers Cox2-Cox5-Cox18-Cox20-Cox22-Cox37-Cox51-Cox56-Cox57-Cox61. This MST genotype has been previously identified colonizing human cardiac valves in France and Germany, goat placenta in Austria (http://ifr48.timone.univ-mrs.fr/mst/coxiella_burnetii/strains.html) and sheep milk products in Tuscany, Italy [24]. The Neighbor-joining tree demostrating the placement of MST 32 (including the *C. burnetii* strain detected in this study) and the phylogenetic relationship with other genotypes, reinforce this zoonotic role (Fig. 2). MST 32 is placed in a tree branch (bootstrap of 70) that comprises several genotypes that particularly affect humans (60 samples) and small domestic ruminants (31 samples), and in a lesser extent cattle (4 samples) (Fig. 2 and Table 1). The data used to produce Fig. 2 are presented at Additional file 1: Table S1.

## Discussion

In Greece, the notification of human Q fever cases is mandatory however under-reporting is one of the main limitations in disease assessment. There is, also, no continuous and reliable recording of the distribution of human and animal cases in the country thereafter, the identification of the source and route of infection remains largely unknown. More than 30 studies have been carried out during the past 20 years in Greece in an attempt to expand the knowledge on *C. burnetii*; they have been focused on the pathogen isolation from humans [25] and the associated disease [26], its detection in ticks [27], on antibiotics resistance [28] and on proteomic analysis [29–31]. Despite the above, very few information is available regarding strain diversity. This study increases knowledge on the subject with the MLVA and MST-typing of nine *C. burnetii* positive samples obtained from different areas of Greece, deciphering its potential zoonotic role.

Overall, three MLVA genotypes (A, B and C) were detected in the current study. Genotype A was the most dominant occurring in different areas of the country, some separated by hundreds of kilometers. This large distribution may be due to the movement of animals from one place to another, or to a common animal supplier (who does not necessarily have to be Greek). Since only sheep samples have been genotyped, it can also be claimed that *C. burnetii* strains circulating in this animal population in Greece are very similar and more/other MLVA loci may be need to increase the discriminatory power. For example, we have failed to amplify the locus *ms26* independently from the specimen and the qPCR Ct values; the reason for this depletion has already been explained in a recent study [32] according to which, the loss of *ms26* is due to a deletion occurring between CBU_0877 and CBU_0881.

The genotypes identified in the current studied were designated as novel ones since no correspondence was found among the loci repeats profiles detected herein and those identified in other European studies, which have been deposited at the open web-site http://mlva.u-psud.fr/MLVAnet/spip.php?rubrique50. Interestingly, the representatives of each of these three MLVA genotypes were found to be identical based on the 10- spacers MST panel and were identified as belonging to MST 32 group when the spacer’s profile was compared with those deposited at http://ifr48.timone.univ-mrs.fr/mst/coxiella_burnetii/strains.html. Up to now the information regarding *C. burnetii* diversity in Greece was restricted to the description of Heizenberg str from an human sample, a strain close related to MST 18 [11]. This study increased the number of *C. burnetii* strains with zoonotic potential which are associated to small ruminants and circulate in the Hellenic territory. MST 32 was previously recorded in human specimens, heart valve (Germany) and aortic biopsy (France), in a goat placenta (Austria) and sheep soft cheese (Italy) [11, 24].

The increased use of molecular methods for epidemiological purposes has proven to be of great value both in the identification of animals seeding *C. burnetii* and tracking-back the disease, especially when accompanied by typing schemes. Since the first proposition of a panel to be used for the genotyping of C. *burnetii* [10, 11], a number of studies have been carried out throughout Europe to increase knowledge on the circulating *strains*. Effort has been made to genotype as many positive samples as possible and to expand the panel used, introducing more loci to increase strain identification. The main focus has been put into MLVA and MST. In fact is has been suggested that MLVA presents the highest discriminatory power [33], while different techniques seem to agree among each other [34, 35]. The great advantage of both methods (but of MLVA in particular) is that they do not require the isolation of the pathogen [10, 11]. Genomic analyses can be made using directly the DNA purified from most type of samples, although results are best achieved if high bacterial load is present, as in the case of aborted material. Furthermore, typing by MLVA can be easily standardized since it has become pretty much straightforward and can be performed without the need of a large budget, and may prove of great usefulness in case of large-scale molecular epidemiology investigations [10]. It has, also, been shown that the selected MLVA markers are of high quality and show considerable stability [36], which is of particulate importance since regions that evolve too rapidly may prove misleading during an epidemiological surveillance [37].

A point of concern regarding MLVA is the presence of PCR products of unexpected size and/or failures to amplify certain loci, an aspect for which little has been discussed. For instance, a study carried out recently has shown that locus ms36 contains the full sequence of locus ms20. Thus, it may be possible that primers targeting ms36 may well amplify both loci resulting in a PCR product whose size may depend on the variations at the 9 bp motif of ms36, the 18 bp motif of ms20 or at both [32]. Further attention is required when analyzing the results to avoid mis-calculations that may lead to erroneous comparisons with data of other studies [38, 39]. A step forward on this has been made through the recent introduction of the microfluidics technology on the field of MLVA typing. A recent study [40] showed that this technology proved to be more reliable and more sensitive when compared to standard electrophoretic techniques, which could add reproducibility, sensitivity and high fidelity to the procedure of typing, making the quality of the results produced being comparable to those of sequencing. Moreover, the lack of harmonization of the MLVA panels and of the loci amplified, allied with the high discriminatory power is leading to results that are difficult to compare among each other [21, 41], which in turn makes sometimes inter-laboratory comparison of results difficult. Thus, it is advantageous to associate MLVA with another typing method, such as MST [36]. As shown here, the selection of this latter method that is less discriminatory and uses standardized nomenclature enables the integration of the results obtained in this study. Adding to the above the use of plasmid characterization in addition to MLVA and MST typing has, also, been proposed [35]. Such an approach may help towards the better deepening on the elucidation of the source of some of the already described genotypes.

In any case, human isolates need to be tested [an attempt towards this direction has already been made by a research group in Hungary [42] using similar techniques in an attempt to identify the genotypes causing disease in humans, their virulence, the different symptoms or their duration that may cause, etc. At the same time, a number of issues need to be resolved when working with human samples, since the human blood may contain much lower DNA concentration compared to animal samples (abortion materials, swabs, etc). In fact, in a study carried out in Croatia [38] the authors failed to amplify by MLVA any of the samples that came out positive by conventional PCR and they attributed this finding to low DNA concentration [7].

To establish the link between the source of *C. burnetii* and a disease case or an outbreak it is crucial to obtain the proper sample. Under this context, abortion material may prove valuable providing information on the genotypic diversity but it constitutes only one approach to tracking-back the *C. burnetii* source. Environmental samples such as surface swabs and aerosols may, also, prove of particular interest [43]. In fact, contaminated aerosols are considered as one of the most important transmission routes for *C. burnetii*, especially when the environmental conditions favor dispersal by aerosol means [6, 44]. Actually, it has been proven that large quantities (> 3 × 10^7^ GE per gram of feces or 10^3^ GE per swab) of the pathogen may remain in faeces and vaginal mucus for more than 2 months [45]. Female ruminants that may suffer from abotrions, also called “superspreaders” [46], release large bacterial burdens into the environment; this aspect together with the viability of the pathogen in litter and manure infected by birth products, makes the need for testing other materials as well, more than necessary. It has, also, been suggested that typing of *C. burnetii* genotypes associated with the wildlife may prove of great importance towards the identification of a potential epidemiological link between wildlife and human and/or livestock cases [47].

In Greece there are no past data because of the absence of any isolates from ruminants over the past decades. Therefore, the current survey constitutes the first attempt to genotype *C. burnetii* strains in Greece. Certainly much more work needs to be done and many more samples need to be tested in order to record as many different genotypes as possible, as well as, to cover most of the country territory. Furthermore, there is certainly a need to compare strains from abortion animals against those of healthy ones. The collection of such data and their comparison with data deposited in international databases will help towards both the continuing of the active surveillance and strain genotyping of the pathogen, as well as, to the better understanding of the epidemiology of the disease across Europe.

## Conclusion

Genotyping of *C. burnetii* in a region is critical when trying to identify the major sources of infection, and to implement efficient farm-based control measures. MLVA analysis has been proven as a great molecular tool, the great advantage of which is that it does not require the isolation of the pathogen.

This is the first report of genotypic diversity among *C. burnetii* strains from Greece. Despite the low number of positive samples tested in our study, we have tried to make the first step on the introduction of genotyping of *C. burnetii* in the country. Certainly, there is much more to do in terms of animal surveillance and of human isolates that need to be genotyped, as well.

## Additional file

Additional file 1: **Table S1.** List of data from studies that have been already carried out in Europe on the aspect *C. burnetii* genotyping. These data were used to build Fig. 2. The corresponding references of the studies are also provided at a separate tab into the file. (XLSX 50 kb)

## Abbreviations

BSL: Biosafety Level
MLVA: Multi-locus variable number of tandem repeats analysis
MST: Multispacer sequence typing
PFGE: Pulse field gel electrophoresis
RFLP: Restriction fragment length polymorphism
SNP: Single nucleotide polymorphism
